# Vision at risk: assessing women’s knowledge of trachoma in Jordan: A cross-sectional survey study

**DOI:** 10.1097/MD.0000000000049766

**Published:** 2026-07-17

**Authors:** Dina M. Abdulmannan, Abdallah Y. Naser

**Affiliations:** a College of Medicine, Umm Alqura University, Makkah, Saudi Arabia; b Department of Applied Pharmaceutical Sciences and Clinical Pharmacy, Faculty of Pharmacy, Isra University, Amman, Jordan.

**Keywords:** eye, females, general public, knowledge, trachoma

## Abstract

Females are at higher risk of being affected with trachoma compared to males as they are the primary caregivers for their children who are one of the main reservoirs for trachoma-related infections. No previous research has assessed women’s knowledge of trachoma in Jordan, which was the purpose of this study. Therefore, the aim of this study was to examine the females’ knowledge of trachoma and its management in Jordan. This was a survey-based online cross-sectional study that was conducted in Jordan between December 27, 2025 and February 1, 2026. The study population for this research comprised of female adults (above 18 years) from the general public in Jordan. This study utilized a previously validated, pretested, structured questionnaire tool that was adapted based on previous research that examined environmental and personal hygiene factors, knowledge about trachoma, and practice toward trachoma prevention. Multivariable logistic regression was performed to assess factors associated with good knowledge (having mean overall knowledge score above 19.56). A total of 535 females were included in the analysis. The mean overall knowledge score was 19.56 ± 5.28, ranging from 10 to 36, with a median of 19. Participants from medical faculties were nearly twice as likely to have good knowledge compared with those from nonmedical faculties (adjusted odds ratio = 1.92, 95% confidence interval: 1.22–3.02; *P* = .005). Additionally, second-year students across all faculties showed significantly higher odds of having good knowledge compared with first-year students (adjusted odds ratio = 1.69, 95% confidence interval: 1.00–2.86; *P* = .049). Regarding treatment and prevention knowledge of trachoma, 237 participants (44.3%) reported eye drops only as the main treatment, while 222 (41.5%) identified antibiotics. Azithromycin was identified by 127 females (23.7%) as commonly used antibiotic in endemic areas. The current study demonstrated inadequate level of awareness of trachoma among the Jordanian women. Being in a medical faculty and in the second year of college were significantly associated with better knowledge of trachoma. Greater efforts must be done to develop effective public health education programs on trachoma, its symptoms and complications, and its management and preventive measures, in order to improve awareness and help in the process of controlling trachoma transmission, especially among first-year, nonmedical faculties students.

## 1. Introduction

Globally, trachoma is the most common infectious cause of blindness, and is caused by an obligatory intracellular bacteria known as *Chlamydia trachomatis*. About 1.9 million people suffer from blindness or visual impairment as a result of this public health issue in 30 different countries.^[1]^ Personal contact through hands, clothing, bedding, or hard surfaces, and flies that have come into contact with an infected person’s eye or nasal discharge can both spread the infection. Trachoma-related blindness is hard to cure; in 2024, 44.4 million people received antibiotic treatment, and 87,349 people had surgery for later stages of the illness.^[1]^

In 1993, the World Health Organization (WHO) adopted the Surgery, Antibiotics, Facial Cleanliness and Environmental Changes (SAFE) strategy, which consists of primary, secondary, and tertiary prevention measures for trachoma, including surgery to prevent blindness in individuals with trichiasis/entropion (tertiary prevention), antibiotics (azithromycin or tetracycline ointment) to fight active chlamydial infection (secondary prevention), and facial hygiene and environmental changes (primary prevention).^[2]^ Trachoma is still one of the most significant health issues in the world, even though the target of eradicating it as a public health issue could be accomplished if the SAFE approach was appropriately implemented.^[3]^

A higher risk of trachoma is linked to a number of conditions including poor environmental sanitation, crowded living conditions, inadequate personal hygiene, and a shortage of water.^[4,5]^ Additionally, the primary factors in the spread and persistence of trachoma infections in communities are inadequate awareness, negative sociocultural attitudes, and insufficient preventive measures.^[6–8]^ In Yemen, a significant proportion of the population lacked knowledge and showed an unfavorable attitude regarding trachoma infection.^[9]^ Nevertheless, good knowledge was significantly associated with a better attitude,^[9]^ highlighting the importance of raising awareness of the disease. Knowledge about trachoma also predicted good practices of trachoma transmission preventive strategies,^[10]^ further underscoring its value in the process of fighting the transmission of this disease.

Females are at higher risk of being affected by trachoma as they are the primary caregivers for their children, who are one of the main reservoirs for trachoma-related infections.^[11–13]^ There is limited research on the prevalence of trachoma in Jordan. Previous research in Jordan reported that the prevalence of trachoma infection among blind patients ranged between 0.04% and 1.03%.^[14,15]^ To the best of our knowledge, no previous research has assessed women’s knowledge of trachoma in Jordan, which was the purpose of this study. The results of this study may inform the development of future awareness campaigns aiming to raise knowledge and awareness of trachoma and its associated risk factors, as well as the effective preventive measures of its spread influencing household hygiene practices, which is an essential part of the WHO SAFE strategy for the prevention of trachoma. Therefore, the aim of this study was to examine females’ knowledge of trachoma and its management in Jordan.

## 2. Methods

### 2.1. Study design

This was a survey-based online cross-sectional study that was conducted in Jordan between December 27, 2025 and February 1, 2026.

### 2.2. Study population and sampling procedure

The study population for this research comprised adult females from the general public in Jordan. Eligible females were invited to participate in this research using the convenience sampling technique. We did not form an exclusion criterion based on participants’ socioeconomic characteristics such as income or education level. The questionnaire link was distributed on social media platforms (Facebook and WhatsApp). The questionnaire link directed the participants to an invitation letter that explained the research’s aim and highlighted the inclusion criteria. We did not offer any incentive for the participants to participate in this research.

### 2.3. Instrument

This study utilized a questionnaire tool that was adapted based on previous research by Yirdaw G. & Tegegne E.^[10]^ The questionnaire tool comprised 19 items and examined females’ knowledge of trachoma and its management. The questionnaire tool collected information on participants’ sociodemographic characteristics (utilizing 7-items of multiple-choice questions format) such as age, education level (their highest academic qualification), occupation (their main source of income), faculty (academic college they are currently enrolled in), level of study (their current academic year in their enrolled program of study), income, and insurance status. The second section examined participants’ knowledge of trachoma (utilizing 12-items of multiple-choice question and yes/no questions format) and asked them whether they have heard about trachoma, where did they hear about it, what information did they hear, causes of trachoma, adverse effects of trachoma, symptoms of trachoma, high risk population of trachoma, whether there is a link between trachoma and animals/livestock, and how can someone protect him or herself from trachoma. Participants were given a score of one for each correct answer. The maximum attainable score was 34, ranging from zero to 34. The higher the score, the better the participants’ knowledge concerning trachoma.

### 2.4. Ethical approval

This study was approved by the Scientific Research Ethics Committee at Isra University, Amman, Jordan (SREC/25/12/180). All participants provided their informed consent before participation.

### 2.5. Statistical analysis

Descriptive statistics such as frequency and percentage were utilized to summarize categorical variables like demographics, while mean, standard deviation, minimum, and maximum were used for continuous variables such as the knowledge score. To assess the normality of the data, the Kolmogorov–Smirnov test was performed. Since the data met the parametric assumptions, independent *t*-tests and 1-way analysis of variance were performed when applicable. Multiple variable logistic regression was performed to assess factors associated with good knowledge (having a mean overall knowledge score above 19.56 ± 5.28). The multiple variable regression model adjusted for participants’ age, education, occupation, faculty of study, level of study, income, and insurance status. Prior to logistic regression, the score was dichotomized into good and poor knowledge based on the mean knowledge score of the study sample. The results were reported as adjusted odds ratio (aOR) and corresponding 95% confidence interval (CI). All analyses were performed using SPSS, version 31 (IBM Corporation [marketed as IBM SPSS Statistics]). The level of significance was 0.05. No missing data were identified in the research dataset. Collinearity diagnostics were checked using variance inflation factor and tolerance measures, which indicated that there was no multicollinearity among the independent variables of the study instrument (the variance inflation factor values ranged between 1.01 and 1.11 and tolerance values ranged between 0.91 and 0.99).

## 3. Results

A total of 535 females were included in the analysis. Most participants were aged 18 to 23 years (467, 87.3%), while 68 (12.7%) were aged 24 to 33 years. Regarding education level, 317 participants (59.3%) held a bachelor’s degree, 125 (23.4%) held a high school education or less. The majority were university students (320, 60.2%), followed by those not working (165, 31.0%). Most participants belonged to nonmedical faculties (233, 72.7%). A total of 414 participants (77.4%) reported a monthly income of < 500, and 306 (57.2%) reported having health insurance (Table 1).

**Table 1:** Sociodemographic characteristics of the study participants.

Sociodemographic characteristics		N (%)
Age (yrs)	18–23	467 (87.3%)
	24–33	68 (12.7%)
Education level	High school or less	125 (23.4%)
	Bachelor	317 (59.3%)
	Diploma	85 (15.9%)
	Postgraduate	8 (1.5%)
Occupation	Not working	165 (31.0%)
	University student	320 (60.2%)
	Medical field	12 (2.3%)
	Not medical field	35 (6.6%)
	Retired	0 (0.0%)
Faculty	Nonmedical	233 (72.7%)
	Medical	87 (27.2%)
Level of study	First year	193 (46.1%)
	Second year	103 (24.6%)
	Third year	53 (12.6%)
	Fourth year	50 (11.9%)
	Fifth year	15 (3.6%)
	Sixth year (for medicine student)	5 (1.2%)
Income (JOD)	< 500	414 (77.4%)
	500–1000	105 (19.6%)
	> 1000	16 (3.0%)
Insurance	No	229 (42.8%)
	Yes	306 (57.2%)

Education level refers to the highest academic qualification, Faculty  refers to the academic college they are currently enrolled in, Level of study  refers to the current academic year in their enrolled program of study.

JOD = Jordanian dinar, N = number of participants.

### 3.1. Trachoma knowledge profile

Overall, correct knowledge about trachoma varied across domains. Approximately half of the participants (243, 45.4%) had heard about trachoma. Most participants reported receiving information from sources other than mass media, volunteers, or health extension workers (355, 66.4%). Correct awareness of preventive measures was relatively high for regular face washing with clean water (390, 72.9%) and maintaining environmental cleanliness (175, 32.7%). Knowledge of transmission and causes was moderate, with 112 participants (20.9%) correctly identifying transmission routes and 164 (30.7%) recognizing causes of trachoma; environmental pollution (215, 40.2%) and washing the face with unclean water (155, 29.0%) were commonly identified causes. Regarding symptoms and complications, itching, swelling, and tearing of the eye (173, 32.3%) and poor vision (148, 27.7%) were the most frequently recognized, although a large proportion reported not knowing adverse effects (362, 67.7%) or symptoms (299, 55.9%). Nearly half of the participants correctly identified trachoma as contagious (246, 46.0%) and acknowledged its link with flies (278, 52.0%) (Table 2).

**Table 2:** The knowledge items and responses.

Item	Response	N (%)
Have you heard about trachoma?*	No	292 (54.6%)
	Yes	243 (45.4%)
Where did you hear about trachoma?*	Mass media	106 (19.8%)
	Trachoma volunteers	58 (10.8%)
	Health extension workers	16 (3.0%)
	Others	355 (66.4%)
What information did you hear about trachoma?		
Causes of trachoma*	Yes	164 (30.7%)
Knowledge of transmission of trachoma*	Yes	112 (20.9%)
Latrine construction and use*	Yes	22 (4.1%)
Face washing*	Yes	59 (11.0%)
Antibiotic treatment*	Yes	50 (9.3%)
Other information*	Yes	320 (59.8%)
Adverse effects of trachoma		
Poor vision*	Yes	148 (27.7%)
Blindness*	Yes	67 (12.5%)
No adverse effects	Yes	36 (6.7%)
Do not know (AE)	Yes	362 (67.7%)
Symptoms of trachoma		
Eye redness*	Yes	130 (24.3%)
Itching, swelling, and tearing of the eye*	Yes	173 (32.3%)
Eye discharge*	Yes	117 (21.9%)
Do not know (symptoms)	Yes	299 (55.9%)
Blurred vision in low light*	Yes	69 (12.9%)
Is trachoma contagious?*	No	289 (54.0%)
	Yes	246 (46.0%)
What makes a person to get trachoma		
Flies as a cause*	Yes	70 (13.1%)
Dirty face*	Yes	146 (27.3%)
Occupation-related cause*	Yes	88 (16.4%)
Direct contact with an infected person*	Yes	156 (29.2%)
Other causes	Yes	283 (52.9%)
Causes of trachoma		
Not washing the face regularly*	Yes	147 (27.5%)
Not using eye medication*	Yes	55 (10.3%)
Washing face with unclean water*	Yes	155 (29.0%)
Sharing a common basin for face washing*	Yes	108 (20.2%)
Environmental pollution*	Yes	215 (40.2%)
Malnutrition*	Yes	19 (3.6%)
Other causes	Yes	227 (42.4%)
Who is most affected by trachoma		
Children under 10 yrs*	Yes	204 (38.1%)
Adolescents (13–18 yrs)*	Yes	181 (33.8%)
Men	Yes	93 (17.4%)
Women	Yes	81 (15.1%)
Other groups	Yes	161 (30.1%)
Is there a link between trachoma and flies?*	No	257 (48.0%)
	Yes	278 (52.0%)
Is there a link between trachoma and animals/livestock?*	No	234 (43.7%)
	Yes	301 (56.3%)
How can someone protect him or herself from trachoma		
Regular face washing with clean water*	Yes	390 (72.9%)
Maintaining environmental cleanliness*	Yes	175 (32.7%)
Surgical intervention*	Yes	60 (11.2%)
Taking antibiotics or medication*	Yes	110 (20.6%)
Use of improved pit latrine*	Yes	66 (12.3%)

AE = adverse effects, N = number of participants.

* Items used to estimate the knowledge score.

The mean overall knowledge score was 19.56 ± 5.28, out of 34, with a median of 19. Participants aged 24 to 33 years had a higher mean score than those aged 18 to 23 years (21.12 ± 5.69) and 19.34 ± 5.19), respectively, *P* = .009. Knowledge scores also differed across the occupation categories, with the maximum mean knowledge score for those working in the medical field (23.92 ± 5.55) (*P* = .03). Students from medical faculties had significantly higher scores than those from nonmedical faculties (20.71 ± 5.42 and 19.14 ± 5.17; *P* = .002). Additionally, students in their sixth year had the highest mean knowledge score and differed significantly from other groups (24.20 ± 7.40) (*P* = .003) (Table 3).

**Table 3:** Sociodemographic characteristics and knowledge score (n = 535).

Variable	Category	Mean ± SD	*P* value
Age (yrs) (n = 535)	18–23	19.34 ± 5.19	.009*
	24–33	21.12 ± 5.69	
Highest education level (n = 535)	High school or less	19.06 ± 4.72	.455**
	Bachelor	19.58 ± 5.34	
	Diploma	20.15 ± 5.66	
	Postgraduate	20.75 ± 7.03	
Occupation (n = 535)	Not working	19.39 ± 5.04	.03**
	Student	19.48 ± 5.27	
	Medical field	23.92 ± 5.55	
	Not medical field	19.63 ± 6.02	
Faculty of study (n = 535)	Nonmedical	19.14 ± 5.17	.002**
	Medical	20.71 ± 5.42	
Level of study (n = 535)	First year	18.30 ± 4.63	.003**
	Second year	20.52 ± 5.41	
	Third year	19.11 ± 5.43	
	Fourth year	19.50 ± 5.25	
	Fifth year	19.20 ± 4.31	
	Sixth year (for medicine student)	24.20 ± 7.40	
Income category (n = 535)	< 500	19.51 ± 5.43	.601**
	500–1000	19.56 ± 4.66	
	> 1000	20.88 ± 5.41	
Insurance status (n = 535)	No	19.34 ± 5.49	.388*
	Yes	19.74 ± 5.13	

ANOVA = analysis of variance, n = number of participants, SD = standard deviation.

* Student *t*-test was used to examine the difference in the mean knowledge score.

** ANOVA test was used to examine the difference in the mean knowledge score.

In the multiple variable logistic regression analysis, faculty type and level of study were associated with good knowledge of trachoma. Participants from medical faculties were nearly twice as likely to have good knowledge compared with those from nonmedical faculties (aOR = 1.92, 95% CI: 1.22–3.02; *P* = .005). Additionally, second-year students showed significantly higher odds of having good knowledge compared with first-year students (aOR = 1.69, 95% CI: 1.00–2.86; *P* = .049). All other variables were not significantly associated with knowledge level (Table 4). The findings of the binary logistic regression analysis are available in the Supplementary Material, Supplemental Digital Content 1, Table S1, Supplemental Digital Content 1.

**Table 4:** Factors associated with good knowledge of trachoma in the multiple logistic regression analysis.

Variables		aOR (95% CI)	*P* value
Age (yrs)	18–23	Reference	
	24–33	1.56 (0.54–4.54)	.411
Education level	High school or less	Reference	
	Bachelor	0.90 (0.43–1.90)	.786
	Diploma	0.84 (0.36–1.95)	.677
	Postgraduate	0.51 (0.07–3.94)	.520
Occupation	Not working	Reference	
	Student	1.76 (0.85–3.63)	.127
	Medical field	3.90 (0.63–24.30)	.144
	Not medical field	0.71 (0.18–2.88)	.635
Faculty of study	Nonmedical	Reference	
	Medical	1.92 (1.22–3.02)	.005
Level of study	First year	Reference	
	Second year	1.69 (1.00–2.86)	.049
	Third year	1.30 (0.67–2.52)	.440
	Fourth year	1.45 (0.73–2.87)	.290
	Fifth year	1.73 (0.57–5.24)	.329
	Sixth year (for medicine student)	1.19 (0.14–10.51)	.874
Income category	< 500	Reference	
	500–1000	1.64 (0.98–2.74)	.060
	> 1000	3.42 (0.82–14.30)	.091
Insurance status	No	Reference	
	Yes	1.00 (0.66–1.51)	.996

The multivariable regression model adjusted for participants’ age, education, occupation, faculty of study, level of study, income, and insurance status.

aOR = adjusted odds ratio, CI = confidence interval.

Regarding knowledge of trachoma treatment and prevention, 237 participants (44.3%) reported eye drops only as the main treatment, while 222 (41.5%) identified antibiotics. Azithromycin was identified by 127 females (23.7%) as a commonly used antibiotic in endemic areas (Table 5).

**Table 5:** Items examined participants’ knowledge of treatment and preventive approaches to trachoma.

Item	Response	
What is the main treatment used to eliminate the infection causing trachoma?	Traditional medicine	39 (7.3%)
	Antibiotics	222 (41.5%)
	Eye drops only	237 (44.3%)
	Painkillers	37 (6.9%)
Which antibiotic is most commonly used to treat trachoma in endemic areas?	Azithromycin	127 (23.7%)
	Amoxicillin	158 (29.5%)
	Penicillin	156 (29.2%)
	Ciprofloxacin	94 (17.6%)
When is surgery recommended for patients with trachoma?	When eyelashes turn inward (trichiasis)	270 (50.5%)
	When there is mild eye redness	46 (8.6%)
	In early mild stages	78 (14.6%)
	Surgery is not used to treat trachoma	141 (26.4%)
Which of the following practices helps prevent recurrence after treatment?	Using cosmetics	29 (5.4%)
	Avoiding sunlight	39 (7.3%)
	Covering the eye	132 (24.7%)
	Regular face washing	335 (62.6%)

## 4. Discussion

The current study aimed to evaluate knowledge about trachoma and its associated factors among women in Jordan. The key findings of this research were that: the current study demonstrated an inadequate level of awareness of trachoma among Jordanian women, participants from medical faculties were nearly twice as likely to have good knowledge compared with those from nonmedical faculties, second-year students across all faculties showed significantly higher odds of having good knowledge compared with first-year students, and a moderate level of knowledge concerning the treatment and prevention of trachoma was identified. This study may help in providing valuable insights for future public educational initiatives aimed at enhancing people’s awareness regarding this disease and its significant consequences if its transmission wasn’t controlled.

### 4.1. Trachoma knowledge

In the current study, participants showed a good level of awareness about preventive practices like regular face washing with clean water (72.9%) and maintaining environmental cleanliness (32.7%). Face washing was generally acknowledged as a household priority in Tanzanian communities where trachoma is widespread, indicating great awareness and a major change in the approach to preventing trachoma transmission over the past few decades.^[16]^ High awareness of face washing has been also reported in rural Ethiopia, where the participants revealed that they were aware of the importance of washing their faces and those of their children on a daily basis.^[17]^ On the other hand, only 20.9% of participants in the current survey correctly identified the routes of transmission, and 30.7% recognized the causes of trachoma, with environmental pollution (40.2%) and washing the face with unclean water (29.0%) being the most commonly identified causes. Additionally, nearly half of the participants in this study knew that trachoma is contagious (46.0%) and acknowledged its link with flies (52.0%). In a study conducted among the Ethiopian community, less than a quarter of the participants correctly recognized contact with discharges, clothes sharing, and fomites as routes of trachoma transmission, whereas 33% correctly identified the cause of this disease.^[18]^ Another survey conducted in Ethiopia revealed that 54.6% of the respondents were aware that trachoma can cause infections among humans, even though awareness about the forms of transmission differs among them, where 24.7% associated it with towels, 35.6% associated it with flies, and 53.1% associated it with filthy fingers.^[19]^ While most participants acknowledged face washing and environmental cleanliness as important, actual routes of transmission were correctly identified by only a minority. This suggests that effective preventive behaviors are based on general hygiene awareness rather than on specific knowledge about trachoma transmission, thus highlighting the need for targeted health education.

Although the majority of the current study’s participants were not knowledgeable of the symptoms (55.9%) and complications (67.7%) of trachoma, approximately a third could correctly identify itching, swelling, and tearing of the eye (32.3%) as symptoms of trachoma, and poor vision (27.7%) as a complication of trachoma. A study conducted in Southern Ethiopia reported inadequate overall knowledge of trachoma among 76.8% of the participants, with only a third identifying itching and tearing of the eye as trachoma symptoms. However, 60% of them could recognize vision loss as a consequence of trachoma.^[18]^ These findings indicate inadequate detailed knowledge of the symptoms and complications of trachoma. Therefore, a greater effort should be applied in targeted health education programs regarding early symptoms and possible complications of trachoma, especially regarding the complication of vision impairment, in order to encourage early treatment-seeking behaviors in the affected individuals, thereby reducing the negative consequences associated with this disease.

### 4.2. Better knowledge associated factors

Participants from medical faculties were almost twice as likely as those from nonmedical faculties to possess good knowledge of trachoma in the current study. According to a study conducted in northern Ethiopia, having ever obtained health education was strongly linked to having a solid understanding of trachoma.^[19]^ Receiving health education was also significantly associated with good knowledge of trachoma in another study.^[20]^ Other studies showed that healthcare faculty students had better knowledge of other infectious diseases such as tuberculosis and coronavirus disease 2019 than their nonmedical counterparts.^[21,22]^ Besides the field of study, students who were in their second year of college had better knowledge than those in their first year as they transitioned from introductory to more applied coursework, which was similar to the results reported in earlier research.^[23]^ Nevertheless, students in their final years, starting from the third year and beyond, did not exhibit a progressive increase in their knowledge, which may appear to be contradictory. This could be due to curriculum saturation, where students in their final years prioritize more specialized or clinical subjects, which could decrease their exposure to other general topics like trachoma. Furthermore, the absence of enduring reinforcement of communicable disease content in advanced coursework may account for the fact that the knowledge advantage observed in second-year students was not maintained in final years. This could imply that the second year may be a critical transitional period in which foundational public health concepts are actively introduced, but are not consistently revisited or deepened in later academic stages. This again highlights the significance of health education programs in raising awareness among the populace about this disease, and the need for medical-related classes to be included and maintained longitudinally in the curriculum, most especially in areas highly endemic with trachoma transmission.

### 4.3. Treatment and prevention

In terms of trachoma treatment and prevention knowledge, 44.3% of participants recognized eye drops as the only primary treatment, whereas 41.5% identified antibiotics. In Kenya, although 83% of the participants identified antibiotic therapy as a modality for trachoma treatment, a very small proportion of them cited eye examination or surgery as trachoma-related care services.^[24]^ Despite the fact that antibiotics like azithromycin and tetracyclines are essential for managing trachoma, participants were unable to recognize antibiotics as the main method of treating the condition, indicating a knowledge gap. As recommended by the WHO, trachoma is treated with azithromycin, which may be taken orally or used as eye drops, or tetracycline eye ointment^[25]^; however, participants identified eye drops as a method of trachoma treatment, without recognizing that it serves as a route of administration to deliver the antibiotic. Moreover, only 23.7% of participants in this study identified azithromycin as the most commonly used antibiotic to treat trachoma in endemic areas. This finding further sheds light on this existing knowledge gap and underscores the urgent need for public health awareness initiatives to improve understanding of the recommended treatment approach for trachoma, including first-line antibiotics, and therapeutic alternatives if the treatment is not successful.

This study has limitations. The cross-sectional online survey study design is not free from limitations as this might restrict its ability to examine causality across the study variables and restrict generalizability to users of social media platforms. Furthermore, this study design is prone to recall bias and sampling bias. Therefore, the study findings should be interpreted carefully.

## 5. Conclusion

The current study demonstrated an inadequate level of awareness of trachoma among Jordanian women. Being in a medical faculty and in the second year of college were significantly associated with better knowledge of trachoma. This could be due to second-year students transitioning from introductory to more applied coursework. Greater efforts must be made to develop effective public health education programs on trachoma, its symptoms and complications, and its management and preventive measures, in order to improve awareness and help in the process of controlling trachoma transmission, especially among first-year, nonmedical faculty students.

## Author contributions

**Conceptualization:** Dina M. Abdulmannan.

**Data curation:** Abdallah Y. Naser.

**Formal analysis:** Abdallah Y. Naser.

**Funding acquisition:** Dina M. Abdulmannan.

**Investigation:** Dina M. Abdulmannan, Abdallah Y. Naser.

**Methodology:** Dina M. Abdulmannan, Abdallah Y. Naser.

**Project administration:** Dina M. Abdulmannan.

**Resources:** Dina M. Abdulmannan, Abdallah Y. Naser.

**Software:** Abdallah Y. Naser.

**Supervision:** Dina M. Abdulmannan.

**Validation:** Dina M. Abdulmannan, Abdallah Y. Naser.

**Visualization:** Dina M. Abdulmannan, Abdallah Y. Naser.

**Writing** – **original draft:** Dina M. Abdulmannan, Abdallah Y. Naser.

**Writing** – **review & editing:** Dina M. Abdulmannan, Abdallah Y. Naser.


