# Supplementary material for: Vision at risk: assessing women’s knowledge of trachoma in Jordan: A cross-sectional survey study
Source: Medicine (Baltimore). 2026 Jul 17;105(29):e49766. doi: 10.1097/MD.0000000000049766 (PMC13384624; doi:10.1097/MD.0000000000049766)
Supplement: Supplementary file 1 [file medi-105-e49766-s001.docx]

Supplementary material:

Table S1: Binary logistic regression analysis

| Variables | | OR (95%CI) | P value |
| --- | --- | --- | --- |
| Age (years) | 18-23 | Reference | |
|  | 24-33 | 1.07 (0.28-4.03) | 0.920 |
| Education level | High school or less | Reference | |
|  | Bachelor | 1.44 (0.98-2.13) | 0.067 |
|  | Diploma | 1.69 (1.00-2.84) | 0.051 |
|  | Postgrad | 2.31 (0.70-7.62) | 0.170 |
| Occupation | Not working | Reference | |
|  | Student | 1.14 (0.80-1.63) | 0.473 |
|  | Medical field | 4.18 (1.46-11.97) | 0.008 |
|  | Not medical field | 1.33 (0.69-2.56) | 0.389 |
| Faculty | Non-medical | Reference | |
|  | Medical | 1.78 (1.25-2.55) | 0.002 |
| Level of study | First year | Reference | |
|  | Second year | 1.92 (1.21-3.05) | 0.006 |
|  | Third year | 1.54 (0.88-2.70) | 0.130 |
|  | Fourth year | 1.85 (1.02-3.36) | 0.043 |
|  | Fifth year | 1.65 (0.61-4.44) | 0.326 |
|  | Sixth year (for medicine student) | 4.63 (0.88-24.44) | 0.071 |
| Income | <500 | Reference | |
|  | 500-1000 | 1.60 (1.08-2.37) | 0.019 |
|  | > 1000 | 2.15 (0.82-5.64) | 0.120 |
| Insurance | No | Reference | |
|  | Yes | 1.28 (0.93-1.77) | 0.131 |
